# The Quality of Life and Medication Adherence in Patients with Multiple Sclerosis—Cross-Sectional Study

**DOI:** 10.3390/ijerph192114549

**Published:** 2022-11-05

**Authors:** Aleksandra Kołtuniuk, Aleksandra Pytel, Dorota Krówczyńska, Justyna Chojdak-Łukasiewicz

**Affiliations:** 1 Department of Nursing and Obstetrics, Faculty of Health Science, Wroclaw Medical University, 51-618 Wroclaw, Poland; 2 Cardinal Stefan Wyszynski Institute of Cardiology, 04-628 Warsaw, Poland; 3 Department of Nursing and Obstetrics, Collegium Mazovia, 08-110 Siedlce, Poland; 4 Department of Neurology, Wroclaw Medical University, 50-529 Wroclaw, Poland

**Keywords:** multiple sclerosis, quality of life, medication adherence, activities of daily living, disease-modifying therapy

## Abstract

Multiple sclerosis (MS) is a chronic, degenerative autoimmune inflammatory disease of the central nervous system. MS is characterized by a wide range of symptoms and unpredictable prognosis, which can severely affect patient quality of life (QOL). The treatment strategy includes acute relapse treatment, disease-modifying treatment (DMT), and symptomatic therapy. Adherence to long-term DMTs is essential in order to maximize the therapeutic effects for MS and is crucial to health-related quality of life (HRQOL). This study aimed to evaluate the relationship between QOL and adherence to DMTs in MS patients. A group of 344 patients (73% females) aged 39.1 years with relapsing-reemitting MS were included. The Multiple Sclerosis International Quality of Life (MusiQOL) and the Multiple Sclerosis Treatment Adherence Questionnaire (MS-TAQ) were used. An injection of interferon (IFN)-β1b was used in 107 patients, IFN-β1a in 94 patients, and glatiramer acetate in 34 patients. The oral treatment includes teriflunomide in 14 patients, dimethyl fumarate in 86 patients, and fingolimod in nine patients. No statistically significant differences (*p* > 0.05) were observed in adherent (ADH) vs. non-adherent patients (non-ADH) in MusiQOL. The total adherence rate was 72% (MS-TAQ). An analysis of the univariate logistic regression model showed an effect of only the activities of daily living (ADL) and relationship with the healthcare system (RHCS) domains on the level of adherence to treatment recommendations. The other variables studied do not affect the level of adherence. Higher QOL levels in the ADL and RHCS domains affect medication adherence in MS patients. Our findings could help manage MS patients, promoting interventions on ADLs and good relationships with healthcare providers to improve their adherence to therapy and result in better QOL.

## 1. Introduction

Multiple sclerosis (MS) is a chronic, autoimmune, and neurodegenerative disease of the central nervous system characterized by inflammatory demyelination. The pathophysiology of MS is based on two main processes: inflammation and neurodegeneration. MS is characterized by neurological symptoms affecting the quality of life (QOL) [1].

While the physical disability aspect of multiple sclerosis (MS) is of paramount importance, quality of life (QoL) measurements are recognized as increasingly important for assessing disease progression, treatment, and the management of care provided to MS patients [2].

Patients with MS have a lower QOL than the general population [3]. QOL in MS can be affected by numerous disease-related factors, such as disability level or the type of MS. Moreover, factors such as coping, mood tone, autonomy, and perceived social support can influence QOL. QOL is also an indicator of treatment success and a predictor of disease progression [1]. Therefore, researchers recommend evaluating the HRQOL as an assessment of the health status as experienced/reported by the patient [4], and it should be included in the definition of No Evidence of Disease Activity [5].

The current treatment strategies for MS include managing acute relapse, disease-modifying therapies (DMTs), and symptomatic treatment. The available DMTs are mostly immunomodulation-based to reduce the inflammatory process and prevent disease progression [4]. Drugs differ in route and frequency of administration, side effects, tolerability, and treatment adherence. Adherence to therapy in chronic disorders is crucial to obtain the benefit of treatment. Moreover, medical adherence in MS is a key factor for successful treatment in long-term treatment [6].

According to the definition, adherence means different aspects of seeking medical attention, acquiring prescriptions, and taking medicines appropriately [7]. Several direct and indirect methods can measure medication adherence. The most common are self-reported questionnaires, structured interviews, or therapeutic drug monitoring. Adherence usually reports the patient’s behavior towards a therapeutic regimen. This includes pharmacological treatment but also non-pharmacological management involving lifestyle or preventive care. Adherence includes a percentage assessment of the level of medications taken over a given period.

Several factors have been associated with therapy adherence, including age, sex, socioeconomic factors, comorbidity, side effects of drug, and MS type. Adherence in MS correlates with lower rates of emergency visits and hospitalization. A low level of adherence is connected with poor outcome and a low quality of life. Adherence to the treatment regimen is the main factor for successful therapeutic response and is connected with higher QOL [8,9,10,11,12]. It was reported that adherent patients had higher scores on the majority of physical and emotional well-being domains of a QOL after 2 months of follow-up [11]. MS patients with higher adherence also had higher mental health [8,9] and pain interference scores on a QOL survey [8].

According to some studies [13,14], adherence to the disease-modifying drugs (DMDs) in MS varies between 41% and 93%. Non-adherence to DMDs for MS is associated with poorer clinical outcomes, including higher relapse rates, disease progression, and an increased frequency of hospitalization [15,16]. However, the relationship between adherence to QOL and other outcomes in Polish patients with MS is unknown.

Therefore, this study aimed to assess the relationship between QOL and adherence to DMTs in MS patients. The primary outcome was to determine adherent and non-adherent MS patients (MS-TAQ) and find differences in the medication adherence in terms of quality of life (MusiQOL). The secondary outcome was to compare adherent and non-adherent patients regarding sociodemographic factors and MS-related clinical factors such as MS type (relapsing-remitting); symptoms (fatigability, dysphagia, hypertonia, paresthesia, and group of disorders: mood, speech, mobility, sexual, vision, sphincter); and pharmacotherapy (type, duration, satisfaction, difficulties). The tertiary outcome was to find independent predictors for non-adherence among sociodemographic and clinical variables, as well as subsequent domains of the QOL.

## 2. Materials and Methods

### 2.1. Study Design and Participants

The investigators used a cross-sectional study design with a questionnaire-based survey. All participants were recruited from patients with MS treated at four leading neurological centers in Wroclaw, Poland. Patients who met the inclusion criteria responded to traditional self-administered pencil-and-paper questionnaires, which were designed to be completed in approximately 15 min during each check-up visit to the neurological center at which they received their DMTs for the next month. Medical data were collected from the hospital database.

The study involved 250 women and 94 men aged between 18 and 69 years who were treated with first and second-line drugs. In the first-line treatment, the following medications were used: IFN-β1a as Avonex (*n* = 48) and Rebif (*n* = 35); pegylated IFN-β1a as Plegridy (*n* = 11); IFN-β1b as Betaferon (*n* = 78) or Extavia (*n* = 29); glatiramer acetate: Copaxone (*n* = 34); teriflunomide: Aubagio (*n* = 14); and dimethyl fumarate: Tecfidera (*n* = 86). The second-line treatment included 9 patients treated with fingolimod (Gilenya). We excluded patients who were treated with natalizumab because IV injections are connected with 100% adherence. The vast majority of respondents lived in a large city (39.1%), were professionally active (71.5%), married (60%), and had a higher education (53.4%). It was demonstrated that 27.9% of the respondents did not follow recommended treatments—patients were identified as non-adherent (non-ADH) if they missed one or more doses of medication in the 28 days prior to completing the survey [17].

### 2.2. Qualification Criteria

The inclusion criteria were (1) a confirmed diagnosis of relapsing-remitting MS (RRMS) based on McDonald Criteria 2010 or 2017 (depending on the time of diagnosis); (2) taking first-line or second-line drugs such as IFN-β1a, pegylated IFN-β1a, IFN-β1b, glatiramer acetate, teriflunomide, dimethyl fumarate and fingolimod; (3) treatment for at least six months prior to participation in the study; (4) a stable MS disease without relapse within 30 days before the study, (5) age over 18; and (6) written informed consent prior to participation in the study.

The exclusion criteria were (1) progressive forms of MS, (2) a confirmed diagnosis of RRMS but not taking the mentioned above first-line or second-line DMTs, (3) patients treated with natalizumab, (4) treatment initiated less than six months before participation in the study, (5) severe cognitive impairment making the patient unable to follow the test instructions, and (6) a lack of written consent to participate in the study.

### 2.3. Ethical Considerations

The study was approved by the Bioethics Committee of the Wroclaw Medical University in Wroclaw, Poland (no. KB-175/2022). All participants were informed of its purpose, timeline, and requirements. They were also informed of the option to withdraw from participation at any stage. All patients provided signed informed consent at the start of the study. The study was carried out following the Declaration of Helsinki and Good Clinical Practice guidelines. The STROBE guidelines (Strengthening the Reporting of Observational Studies in Epidemiology) were followed due the cross-sectional study design.

### 2.4. Research Instruments

Data collection and measurement tools used in this study included a sociodemographic and clinical survey and two standardized questionnaires: the Polish version of the Multiple Sclerosis International Quality of Life Questionnaire (MusiQOL) [18] and the Polish version of the Multiple Sclerosis Treatment Adherence Questionnaire (MS-TAQ) [19].

#### 2.4.1. Survey

The authors designed a semi-structured and self-administered questionnaire for sociodemographic data (e.g., age, sex, place of residence, education level, marital, and financial status) and clinical data (MS type and duration and type of pharmacotherapy).

#### 2.4.2. Multiple Sclerosis International Quality of Life Questionnaire (MusiQOL)

The MusiQOL is a research tool aimed at assessing the QOL of MS patients. It contains 31 questions concerning the patient’s life during the last four weeks, with the following verbal answers: never, rarely, sometimes, often, always, and not applicable. The MusiQOL questionnaire makes it possible to assess the QOL of MS patients in 10 domains: ADL—activities of daily living; PWB—psychological well-being; RFr—relationships with friends; SPT—symptoms; RFa—family relationships; RHCS—satisfaction with the healthcare system; SSL—sentimental and sexual life; COP—coping; REJ—rejection; and Total—overall QOL. The QOL in each domain is expressed by a number ranging from 0 to 100 (a higher score indicates better QOL). No norms exist in the case of MusiQOL, and as such, it is impossible to say whether the respondents’ results indicate high or low QOL; one can only compare the individual domains with each other to identify the areas of high and low QOL. The scale dimensions exhibited high internal consistency (Cronbach’s alpha between 0.67 and 0.90 for the Polish version) [18].

#### 2.4.3. Multiple Sclerosis Treatment Adherence Questionnaire (MS-TAQ)

The MS-TAQ questionnaire is a self-administered tool for identifying barriers to MS- patient adherence to a prescribed DMT regimen. The questionnaire consists of 30 questions, which are categorized into three subscales. DMT-Barriers (DMT-BARR) assesses the importance (on a four-point scale ranging from “not important at all” to “extremely important”) of 13 barriers to adherence in MS patients who have missed at least one dose in the previous 28 days. DMT-Side Effects (DMT-SE) describes the frequency (on a five-point scale from “never” to “all or nearly all of the time") of ten side effects. This was asked of all patients. DMT-Coping Strategies (DMT-COPE) assesses seven coping strategies used by patients to reduce side effects (e.g., using an ice cube on the injection site). This was asked of all patients and had a binary yes/no response for “in the past four weeks did you usually.”) [17]. The Polish version of MS-TAQ has high reliability; Cronbach’s alpha coefficient is 0.57 for DMT-COPE, 0.89 for DTM-BARR, and 0.90 for DTM-SE [19].

### 2.5. Statistical Analyses

The statistical analysis was performed using Statistica software version 13.0 (StatSoft, Dell Inc., Tulsa, OK, USA). For the measurable variables, the arithmetic mean (M), median (Me), standard deviation (SD), and extreme values (Min and Max) were calculated; for the non-measurable variables, the percentages (%) were calculated. All quantitative variables were tested using the Shapiro–Wilk test to determine their type of distribution. The nonparametric Mann–Whitney U test was used to compare the results between groups: adherent (ADH) vs. non-adherent (non-ADH) for continuous variables, and the chi-squared test was used for categorical data. A forward-stepwise univariate-logistic regression analysis was used to identify the factors associated with adherence to recommended treatments. It was assumed that variables associated with adherence (*p*  <  0.30) would be included in the multifactorial model. For all comparisons, the level of α  =  0.05 was assumed.

## 3. Results

Table 1 presents the sociodemographic characteristics of the study group, including non-adherent and adherent MS patients. No statistically significant difference due to sociodemographic variables and the degree of adherence to treatment recommendations was noted (Table 1).

A comparison was also made between the adherence and non-adherence groups due to clinical variables and opinions about the treatment process. Statistically significant differences (*p* < 0.05) were observed due to the type of multiple sclerosis medication taken. The non-adherence group took Betaferon the most (29.1%; *n* = 28), while the adherence group used Tecfidera the most (25.0%; *n* = 62) (Table 2). In addition, there were statistically significant differences (*p* < 0.05) in the frequency of medication in the last 28 days. The non-adherence group was more likely to use the drug every other day than the adherence group (38.5%; *n* = 37 vs. 28.2%; *n* = 70). Statistically significant differences also occurred in the frequency of injections by others (*p* < 0.05). In other cases, the results were not statistically significantly different (*p* > 0.05) (Table 2).

Table 3 shows a comparison of the incidence of symptoms in the adherence and non-adherence patient groups. Statistically significant differences in results (*p* < 0.05) were observed for dysphagia. Among the non-adherence group, dysphagia occurred in 8.3% (*n* = 8) of patients, and in the adherence group, only in 3.2% (*n* = 8) of patients. Otherwise, no statistically significant differences (*p* > 0.05) were observed (Table 3).

The results of the MusiQOL questionnaire were also compared between the study groups. No statistically significant differences (*p* > 0.05) were observed (Table 4) in the group of non-adherent and adherent patients by QoL domains.

An assessment of the association of variables such as sex, place of residence, education, marital status, professional activity, complaints (fatigability, dysphagia, speech disorders, hypertonia, mood disorders, paresthesia, neuralgia, mobility and balance disorders, sexual disorders, visual disorders, sphincter disorders), and treatment satisfaction and QOL levels in each domain was conducted. An analysis of the univariate logistic regression model showed an effect of only the ADL and RHCS domains on treatment adherence. The other variables studied did not affect the level of adherence (Table 5).

## 4. Discussion

MS management aims to reduce the frequency of relapses, slow disease progression, and improve QOL. Adherence is an important issue in the management of MS patients [20]. Poor adherence is linked with an increased risk of relapse and MS-related hospitalizations [21]. Discontinuation of DMT therapy has been associated with an increased level of instability. Many factors have an impact on lower adherence to DMT (medication tolerability, disease duration, frequency and method of drug dosing, and treatment duration).

Our results demonstrate a total adherence rate of 72%, which is similar to other research [22]. Based on the results of previous studies, the adherence rate in MS patients ranges between 41–88% [13]. For example, in the cross-prospective Canadian study [20], one in five participants reported missing more than 20% of their doses of medication, and less than half were fully adherent in the last 30 days [23].

The present study did not confirm that sociodemographic factors might affect adherence. In contrast, previous reports have shown that lower levels of education or lower socioeconomic classes significantly affect poor adherence. A study by Järvinen et al. [24] found that higher adherence levels were observed in women and younger patients. In contrast, in a study by Cerghet et al. [8], higher adherence was associated with an increased likelihood of employment. Interestingly, Devonshire et al. [10] and Duchovskiene et al. [12] showed that patients with higher education were less likely to adhere to recommendations.

Previous studies have compared patients’ adherence to different MS medications [10,15,25,26]. The most common causes of this non-adherence were forgetfulness and injection-related factors (injection fatigue, fear of injection, and injection-related side effects). Other causes included: the frequency of drug administration, secondary effects inherent to the medication, and the patient’s perception of drug efficiency [27]. Bergvall et al. [28] have shown that adherence to oral medication is higher than to injectable and infusible DMTs.

Devonshire and al [10] showed that the adherence rates for the injectable drugs ranged from 87% (i.m. IFN-β1a) to 65% (GA) during the long-term therapy. In the study by Halpern et al. [25], the intramuscular IFN-β1a had significantly higher adherence odds than other DMT cohorts during the 8-year period. In a German retrospective cohort study [26], only 30–40% of patients with MS were adherent to self-injected DMT therapy two years after initiation of treatment [29]. Our results showed that among injectable drugs, patients treated with Plegridy and Avonex were more adherent than patients on other DMTs (100% (Plegridy)/90% (Avonex) vs. 71% (Rebif)/68% (GA)/69% (Extavia)/64% (Betaferon).

Ozura et al. [26] suggest that better adherence in the case of injectable therapy is connected with the frequency of DMT administration. Arroyo et al. [15] showed a higher adherence rate in treatment with Avonex than with the rest of the medications. In our study, patients who used injectable medication every other day (IFN-β1b—Betaferon) were more non-adherent.

The literature on adherence to injectable DMTs for patients with MS suggests that improvements in administration, including the increased ease of administration by using auto-injectors, could improve adherence to treatment. In our study, MS patients who used self-injection often were more adherent than non-adherent patients.

Arroyo et al. [15] showed that the adherence rate in the group of oral drugs was 78% for fingolimod, 72% for dimethyl fumarate, and 50% for teriflunomide. According to previous studies, adherence to fingolimod treatment has been higher than other oral DMTs [28,30]. The meta-analysis by Nicholas et al. [31] demonstrated that one in five patients do not adhere to once- or twice-daily oral DMDs, and one in four patients discontinue the initially prescribed oral DMDs before one year. In our study, one in four patients was non-adherent to dimethyl fumarate, one in five patients was non-adherent to fingolimod, and every second patient was non-adherent to teriflunomide, which is in line with the results of recent evidence.

Many studies [8,32,33,34,35] have shown that a higher disability score on the Expanded Disability Status Scale (EDSS) is connected with a lower level of adherence to therapeutic recommendations and a higher rate of treatment discontinuation. In our study, patients with a better QOL in the ADL domain were more likely to be non-adherent. McKay et al. [23] have also shown that persons with mild disabilities (in EDSS) are less likely to be adherent. This may be because patients with MS, who are more active in daily life (have a higher QoL in the ADL domain), are less motivated to use the drugs regularly.

The relationship between a patient and their doctors plays a crucial role in the treatment process [36]. Patients with MS, who are active in the decision-making process, are more adherent to treatment plans [37]. Camara et al. [36] reported that receiving information about the disease, contacting the doctor more often, and not missing check-up visits contribute to treatment adherence. Adequate care, education, and a specific program, i.e., Continuous Care Model (CCM) using a smartphone application, are the factors responsible for high adherence among patients with MS [38,39]. In our study, patients with a higher QOL in the RHCS domain (more satisfied with the healthcare system) adhered more to the treatment recommendation.

Adherence and QOL are connected and significantly affect patient management and care [40]. Several studies [41,42,43,44] of chronic illnesses have shown that adherent patients had improved QOL. In our study, there was no statistical difference in QOL between both groups (adherent (ADH) versus non-adherent (non-ADH)), which was also noted by Duchovskiene et al. [12]. Studies conducted among patients with MS showed that a higher QOL score was a significant predicting factor for higher adherence [10,11,12,45]. In our study, only two domains (ADL and RHCS) significantly affected treatment adherence.

### 4.1. Clinical Implications

Understanding the factors that affect MS patient adherence to recommended treatments allows those treatments to be planned as effectively as possible, but aids in detecting when a patient fails to adhere to treatment. The well-being of a patient is one of the most crucial factors that can affect their adherence and prognosis. Reduced QOL in MS patients is linked to motivation to adhere to a DMT. It is crucial to emphasize that the good physical condition (low EDSS) of MS patients results from taking regular medication. In particular, when patients feel well and are active in their daily lives, they may forget to take their medications regularly.

Previous studies have shown that patients more preferably use oral pharmacotherapy [46,47,48]. Therefore, the level of adherence among patients taking oral drugs is higher than those taking injectable drugs. Our study did not confirm this relationship. Nevertheless, a trend was revealed (no statistical significance) showing that those patients who administer the drug via injection (every second day) are statistically significantly more likely to be non-adherent. On the other hand, individuals who inject drugs at wider intervals are more likely to be adherent. The fact is that when choosing a medication, it is worth consulting with the patient about the form of the drug and, consequently, its method and frequency of administration.

The healthcare providers managing MS patients who undergo immunomodulatory treatment should constantly measure patient-reported outcomes, e.g., QOL, the level of depression, and anxiety, to improve their level of adherence and, in the next step, to improve their health status. Good communication skills with healthcare providers (self-efficacy in communication with healthcare providers) and adequate care are linked to medication adherence. The medical team should also offer informational and emotional support throughout any treatment. The relationship between a patient and their healthcare team is significant because patients who are more satisfied with care (with higher QoL in RHCS) better adhere to their treatment plan.

Moreover, it should be emphasized that in Poland, the healthcare system is free of charge. Therefore, all insured persons who meet the criteria for inclusion in the drug program receive medication. However, the waiting time for qualification into treatment is relatively long, and the inclusion criteria are quite restrictive. Therefore, it is estimated that only 13% of MS patients in Poland receive the drugs. Hence, it is particularly important to monitor adherence in those receiving treatment, ensuring that funds spent on this purpose are not wasted.

### 4.2. Study Limitations

Although our study was carefully designed, a few limitations should be mentioned. One is that data on the subjective assessment of adherence to recommended treatments came from a single standardized questionnaire (MS-TAQ). Another is that the assessment of QOL was made based on the subjective opinion of the patients themselves. Moreover, the cross-sectional nature of the data precludes any consideration of potential causal relationships between variables, so further research is needed to test this aspect. Finally, the study was conducted in one clinical and academic center in Wroclaw, and the results should not be directly generalized to other populations. Nevertheless, it should be emphasized that a substantial number of patients treated were from different parts of Lower Silesia and the southwestern region of Poland, rather than a single city. Nevertheless, multicenter studies are still needed.

## 5. Conclusions

To our knowledge, this is the first study in Poland assessing the connection between adherence and QOL. Our results highlight that a majority of MS patients were adherent to treatment. The adherence level does not differentiate MS patients regarding QOL. A higher level of QOL in the ADL and RHCS domains affects medication adherence in MS.

## Figures and Tables

**Table 1:** Characteristics of the study group with a comparison between adherence and non-adherence groups.

Study Group (*n* = 344)								
Variables		Total		Group				*p*-Value *
				Non-ADH (*n* = 96)		ADH (*n* = 248)		
		*n*	%	*n*	%	*n*	%	
Sex	Female	250	72.7	69	71.9	181	73.0	0.836
	Male	94	27.3	27	28.1	67	27.0	
Place of residence	Village	86	25.1	29	30.2	57	23.1	0.574
	City <100,000 inhabitants	42	12.2	11	11.5	31	12.6	
	City 100–500,000 inhabitants	81	23.6	20	20.8	61	24.7	
	City >500,000 inhabitants	134	39.1	36	37.5	98	39.7	
Education	Basic or vocational	42	12.2	9	9.4	33	13.4	0.600
	Secondary	118	34.4	34	35.4	84	34.0	
	Higher	183	53.4	53	55.2	130	52.6	
Marital status	Single	128	37.3	32	33.3	96	38.9	0.754
	Married	206	60.0	62	64.7	144	58.3	
	Divorced	4	1.2	1	1.0	3	1.2	
	Widowed	5	1.5	1	1.0	4	1.6	
Professional activity	Student	16	4.7	5	5.2	11	4.4	0.136
	Employed	246	71.5	77	80.2	169	68.2	
	Disability	55	16.0	11	11.5	44	17.8	
	Retirement pension	10	2.9	1	1.0	9	3.6	
	Unemployed	17	4.9	2	2.1	15	6.0	
**Variables**		**M**	**SD**	**M**	**SD**	**M**	**SD**	*p*-Value **
Age		39.1	10.0	38.0	8.7	39.6	10.4	0.18

*n*, number of participants; %, percent of participants; * χ^2^ test; ** *t*-test.

**Table 2:** The comparison regarding the MS type and treatment used in adherent and non-adherent patients.

Study Group (*n* = 344)								
Variables		Total		Group				*p*-Value *
				Non-ADH (*n* = 96)		ADH (*n* = 248)		
		n	%	n	%	n	%	
Disease duration	1 year	10	2.9	2	2.1	8	3.2	0.109
	2–5 years	86	25.0	18	18.8	68	27.4	
	6–10 years	118	34.3	42	43.7	76	30.6	
	>10 years	130	37.8	34	35.4	96	38.8	
Clinical type of MS	Relapsing-remitting	344	99.4	96	100.0	248	100	0.378
DMDs	Tecfidera (dimethyl fumarate)	86	25.0	24	25.0	62	25.0	**0.021**
	Plegridy (peginterferon β1a)	11	3.2	0	0.0	11	4.4	
	Avonex (IFN-β1a i.m.)	48	14.0	5	5.2	43	17.3	
	Rebif (IFN-β1a)	35	10.2	10	10.4	25	10.1	
	Copaxone (glatiramer acetate)	34	9.9	11	11.5	23	9.3	
	Aubagio (teriflunomide)	14	4.1	7	7.3	7	2.8	
	Betaferon (IFN-β1b—s.c.)	78	22.7	28	29.1	50	20.2	
	Gilenya (fingolimod)	9	2.6	2	2.1	7	2.8	
	Extavia (IFN-β1 b—s.c.)	29	8.3	9	9.4	20	8.1	
How many days during the last 4 weeks (28 days) were you supposed to take this medication?	Twice daily (56 times)	86	25.0	24	25.0	62	25.0	**0.008**
	Every day (28 times)	23	6.7	9	9.4	14	5.6	
	Every other day (14 times)	107	31.1	37	38.5	70	28.2	
	Three times a week (12 times)	69	20.1	21	21.9	48	19.4	
	Once a week (4 times)	48	14.0	5	5.2	43	17.4	
	Twice a month	11	3.1	0	0.0	11	4.4	
During the past 4 weeks (28 days) did you manually inject, use an auto-injection device, or do both?	Self-injection only	158	67.2	34	53.9	124	72.1	**0.026**
	Manual injection only	66	28.1	24	38.1	42	24.4	
	Both manual injection and self-injection	11	4.7	5	7.9	6	3.5	
During the past 4 weeks (28 days), how often was your injection done by someone else?	Never	163	69.4	50	79.4	113	65.7	0.074
	Several times	27	11.5	4	6.4	23	13.4	
	About half of the time	12	5.1	5	7.9	7	4.1	
	In most cases	4	1.7	0	0.0	4	2.3	
	Always or almost always	29	12.3	4	6.4	25	14.5	
Overall, how difficult or easy is it to take your currently prescribed drug for MS?	Extremely difficult	2	0.6	1	1.0	1	0.4	0.670
	Very difficult	12	3.5	4	4.2	8	3.2	
	Moderately difficult	69	20.1	15	15.6	54	21.8	
	Somewhat easy	49	14.2	13	13.6	36	14.5	
	Extremely easy	212	61.6	63	65.6	149	60.1	
Overall, how satisfied are you with the current treatment within the past 4 weeks (28 days)?	Fully satisfied	100	29.1	32	33.3	68	27.4	0.366
	Very satisfied	109	31.7	23	24.0	86	34.7	
	Moderately satisfied	90	26.2	27	28.1	63	25.4	
	Somewhat satisfied	26	7.5	7	7.3	19	7.7	
	Not satisfied at all	19	5.5	7	7.3	12	4.8	

*n*, number of participants; %, percent of participants; * χ^2^ test, in bold—value statistically significant (*p* < 0.05).

**Table 3:** Comparison of the incidence of symptoms in adherent and non-adherent patients.

Study Group (*n* = 344)									
Variables			Total		Group				*p*-Value *
					Non-ADH (*n* = 96)		ADH (*n* = 248)		
			*n*	%	*n*	%	*n*	%	
Complains	Fatigability	No	121	35.2	40	41.7	81	32.7	0.117
		Yes	223	64.8	56	58.3	167	67.3	
	Dysphagia	No	328	95.3	88	91.7	240	96.8	**0.044**
		Yes	16	4.7	8	8.3	8	3.2	
	Speech disorders	No	317	92.2	89	92.7	228	91.9	0.811
		Yes	27	7.8	7	7.3	20	8.1	
	Hypertonia	No	245	71.2	64	66.7	181	73.0	0.246
		Yes	99	28.8	32	33.3	67	27.0	
	Mood disorders	No	241	70.1	70	72.9	171	69.0	0.471
		Yes	103	29.9	26	27.1	77	31.0	
	Paresthesia, Neuralgia	No	255	74.1	76	79.2	179	72.2	0.184
		Yes	89	25.9	20	20.8	69	27.8	
	Mobility and balance disorders	No	154	44.8	49	51.0	105	42.3	0.145
		Yes	190	55.2	47	49.0	143	57.7	
	Sexual disorders	No	250	72.7	70	72.9	180	72.6	0.950
		Yes	94	27.3	26	27.1	68	27.4	
	Vision disorders	No	204	59.3	59	61.5	145	58.5	0.613
		Yes	140	40.7	37	38.5	103	41.5	
	Sphincter disorders	No	238	69.2	65	67.7	173	69.8	0.712
		Yes	106	30.8	31	32.3	75	30.2	

*n*, number of participants; %, percent of participants; * χ^2^ test, in bold—value statistically significant (*p* < 0.05).

**Table 4:** Comparison of the results of the MusiQOL questionnaire between adherent and non-adherent patients.

Domains	Group														
	Non-ADH (*n* = 96)							ADH (*n* = 248)							*p*-Value *
	M	Me	Min	Max	Q1	Q3	SD	M	Me	Min	Max	Q1	Q3	SD	
ADL	70.2	75.0	0.0	100.0	53.1	90.6	23.8	64.2	68.8	0.0	100.0	46.4	87.5	26.1	0.062
PWB	60.7	62.5	0.0	100.0	40.6	81.3	25.3	59.7	62.5	0.0	100.0	43.8	75.0	23.8	0.695
RFr	49.4	50.0	0.0	100.0	25.0	75.0	31.7	43.5	41.7	0.0	100.0	25.0	66.7	28.5	0.143
SPT	72.4	75.0	18.8	100.0	56.3	93.8	20.5	68.5	68.8	12.5	100.0	50.0	87.5	22.2	0.165
RFC	46.7	50.0	0.0	100.0	8.3	75.0	37.0	40.9	33.3	0.0	100.0	8.3	75.0	34.9	0.214
RHSC	59.7	66.7	0.0	100.0	41.7	87.5	32.5	67.6	75.0	0.0	100.0	50.0	91.7	27.3	0.087
SSL	49.6	50.0	0.0	100.0	25.0	75.0	34.5	44.7	37.5	0.0	100.0	25.0	62.5	32.4	0.254
COP	58.5	50.0	0.0	100.0	37.5	87.5	29.7	59.4	62.5	0.0	100.0	37.5	87.5	30.0	0.726
REJ	84.5	100.0	0.0	100.0	75.0	100.0	23.7	80.3	100.0	0.0	100.0	62.5	100.0	25.7	0.178
MusiQOL—total score	61.6	61.5	28.9	97.9	52.2	72.7	13.7	58.8	56.8	24.3	97.2	49.8	68.0	14.6	0.103

*n*, number of participants; M, mean; Me, median; Min, minimum; Max, maximum; Q1, lower quartile; Q3, upper quartile; SD, standard deviation; ADL, activities of daily living; PWB, psychological well-being; RFr, relationships with friends; SPT, symptoms; RFa, family relationships; RHCS, satisfaction with the healthcare system; SSL, sentimental and sexual life; COP, coping with the disease; REJ, rejection; * Mann–Whitney U test.

**Table 5:** Results of the logistic regression.

Adherent/Non-Adherent (Modeled Probability: Non-ADH)							
Variables		Regression Coefficients (B)	Standard Error	*p*-Value	Odds Ratio	95% CI Lower	95% CI Upper
Age		−0.02	0.01	0.18	0.98	0.96	1.01
Sex	Female						
	Male	0.06	0.27	0.84	1.06	0.62	1.79
Place of residence	Village						
	City <100,000 inhabitants	−0.36	0.42	0.39	0.70	0.31	1.58
	City 100–500,000 inhabitants	−0.44	0.34	0.20	0.64	0.33	1.27
	City >500,000 inhabitants	−0.33	0.30	0.28	0.72	0.40	1.30
Education	Basic or vocational education						
	Secondary education	0.39	0.43	0.36	1.48	0.64	3.43
	Higher education	0.40	0.41	0.33	1.49	0.67	3.34
Marital status	Single						
	Married	0.26	0.25	0.31	1.29	0.78	2.13
	Divorced	0.00	1.17	1.00	1.00	0.10	9.96
	Widowed	−0.29	1.14	0.80	0.75	0.08	6.96
Professional activity	Student						
	Employed	0.00	0.56	1.00	1.00	0.34	2.98
	Disability	−0.60	0.64	0.35	0.55	0.16	1.91
	Retirement pension	−1.41	1.18	0.23	0.24	0.02	2.49
	Unemployed	−1.23	0.93	0.19	0.29	0.05	1.80
Fatigability	No						
	Yes	−0.39	0.25	0.12	0.68	0.42	1.10
Dysphagia	No						
	Yes	1.00	0.52	0.052	2.73	0.99	7.49
Speech disorders	No						
	Yes	−0.11	0.46	0.81	0.90	0.37	2.19
Hypertonia	No						
	Yes	0.30	0.26	0.25	1.35	0.81	2.25
Mood disorders	No						
	Yes	−0.19	0.27	0.47	0.82	0.49	1.39
Paresthesia, Neuralgia	No						
	Yes	−0.38	0.29	0.19	0.68	0.39	1.20
Mobility and balance disorders	No						
	Yes	−0.35	0.24	0.15	0.70	0.44	1.13
Sexual disorders	No						
	Yes	−0.02	0.27	0.95	0.98	0.58	1.67
Vision disorders	No						
	Yes	−0.12	0.25	0.61	0.88	0.54	1.43
Sphincter disorders	No						
	Yes	0.10	0.26	0.71	1.10	0.66	1.83
In general, how satisfied are you with the current treatment within the past 4 weeks (28 days)?	Fully satisfied						
	Very satisfied	−0.57	0.32	0.076	0.57	0.31	1.06
	Moderately satisfied	−0.09	0.31	0.767	0.91	0.49	1.69
	Somewhat satisfied	−0.25	0.49	0.618	0.78	0.30	2.05
	Not satisfied at all	0.22	0.52	0.681	1.24	0.45	3.45
ADL		0.01	0.00	**0.049**	1.01	1.01	1.02
PWB		0.00	0.00	0.71	1.00	0.99	1.01
RFr		0.01	0.00	0.10	1.01	1.00	1.01
SPT		0.01	0.01	0.14	1.01	1.00	1.02
RFC		0.00	0.00	0.19	1.00	1.00	1.01
RHSC		−0.01	0.00	**0.027**	0.99	0.98	1.00
SSL		0.00	0.00	0.24	1.00	1.00	1.01
COP		0.00	0.00	0.81	1.00	0.99	1.01
REJ		0.01	0.01	0.18	1.01	1.00	1.02
MusiQOL—total score		0.01	0.01	0.11	1.01	1.00	1.03

OR, odds ratio; CI, confidence interval, in bold—value statistically significant (*p* < 0.05).

## Data Availability

The data presented in this study are available on request from the corresponding author.
